# Exposure to benzene at work and the risk of leukemia: a systematic review and meta-analysis

**DOI:** 10.1186/1476-069X-9-31

**Published:** 2010-06-28

**Authors:** Abdul Khalade, Maritta S Jaakkola, Eero Pukkala, Jouni JK Jaakkola

**Affiliations:** 1 Institute of Occupational and Environmental Medicine, University of Birmingham, UK; 2 Center for Environmental and Respiratory Health Research, Respiratory Medicine Unit, Department of Internal Medicine, Institute of Clinical Medicine, University of Oulu, P.O. B. 5000, 90014 Oulu, Finland; 3 Finnish Cancer Registry, Institute for Statistical and Epidemiological Cancer Research, Pieni Roobertinkatu 9, Helsinki, Finland; 4 School of Public Health, University of Tampere, Tampere, Finland; 5 Center for Environmental and Respiratory Health Research, Institute of Health Sciences, University of Oulu, P.O. B. 5000, 90014 Oulu, Finland

## Abstract

**Background:**

A substantial number of epidemiologic studies have provided estimates of the relation between exposure to benzene at work and the risk of leukemia, but the results have been heterogeneous. To bridge this gap in knowledge, we synthesized the existing epidemiologic evidence on the relation between occupational exposure to benzene and the risk of leukemia, including all types combined and the four main subgroups acute myeloid leukemia (AML), acute lymphocytic leukemia (ALL), chronic lymphocytic leukemia (CLL), and chronic myeloid leukemia (CML).

**Methods:**

A systematic literature review was carried out using two databases 'Medline' and 'Embase' from 1950 through to July 2009. We selected articles which provided information that can be used to estimate the relation between benzene exposure and cancer risk (effect size).

**Results:**

In total 15 studies were identified in the search, providing 16 effect estimates for the main analysis. The summary effect size for any leukemia from the fixed-effects model was 1.40 (95% CI, 1.23-1.57), but the study-specific estimates were strongly heterogeneous (I^2 ^= 56.5%, Q stat = 34.47, p = 0.003). The random-effects model yielded a summary- effect size estimate of 1.72 (95% CI, 1.37-2.17). Effect estimates from 9 studies were based on cumulative exposures. In these studies the risk of leukemia increased with a dose-response pattern with a summary-effect estimate of 1.64 (95% CI, 1.13-2.39) for low (< 40 ppm-years), 1.90 (95% CI, 1.26-2.89) for medium (40-99.9 ppm-years), and 2.62 (95% CI, 1.57-4.39) for high exposure category (> 100 ppm-years). In a meta-regression, the trend was statistically significant (P = 0.015). Use of cumulative exposure eliminated heterogeneity. The risk of AML also increased from low (1.94, 95% CI, 0.95-3.95), medium (2.32, 95% CI, 0.91-5.94) to high exposure category (3.20, 95% CI, 1.09-9.45), but the trend was not statistically significant.

**Conclusions:**

Our study provides consistent evidence that exposure to benzene at work increases the risk of leukemia with a dose-response pattern. There was some evidence of an increased risk of AML and CLL. The meta-analysis indicated a lack of association between benzene exposure and the risk of CML.

## Background

Le Noire and Claude published in 1897 the first report on the possible role of occupational exposure to benzene in the development of leukemia [1]. Since then a substantial number of epidemiologic studies in different occupational groups have assessed benzene exposure and made attempts to quantify the magnitude of risk related to such exposure. In 2005, Schnatter and colleagues published a systematic review of the available 22 epidemiologic studies of the relation between benzene exposure and leukemia subtypes [2]. They concluded that there was consistent evidence that the risk of acute myeloid leukemia (AML) is related to benzene exposure with an indication of a dose-response pattern, and a suggestion for chronic lymphoid leukemia (CLL), whereas the data for chronic myeloid leukemia (CML) and acute lymphocytic leukemia (ALL) are sparse. They did not present any quantitative assessment of these relations. To our knowledge there are no previous meta-analyses that have estimated the effect of exposure to benzene on the risk of leukemia taking into account the cumulative exposure from individual studies. To bridge this gap in current knowledge, we synthesized the existing epidemiologic evidence on the relation between occupational exposure to benzene and the risk of any leukemia and the risks of main subtypes of leukemia in adults, including AML, ALL, CLL, and CML.

## Methods

### Search strategy and inclusion criteria

We conducted a systematic literature review using Medline and Embase databases from 1950 through July 2009. The following search terms were applied: benzene [Benzene derivatives, Polycyclic aromatic hydrocarbons]; occupational exposure, [Inhalation exposure, Maximum allowable concentration, Threshold limit values] and cancer [Neoplasms]. The search command was further refined to include any leukemia combined [leukemia, lymphoid] and the subgroups of leukemia, including AML, CML, and CLL. The Newcastle-Ottawa-Scale (NOS) was used to assess the quality of papers. The articles from the search were then screened according to the following *a priori *inclusion criteria:

(1) Provides information that can be used to estimate the relation between benzene exposure and cancer risk (effect size) in terms of odds ratio (OR), relative risk (RR), standardized mortality ratio (SMR), standardized relative risk (SRR), cumulative incidence ratio (CIR), or standardized incidence rate ratio (SIR);

(2) Original study;

(3) Provides comparable measures of effect estimates and/or cumulative exposure to benzene

(4) Is a cohort, case-control or cross-sectional study in design; and

(5) Includes occupationally active adults as a study population.

The selection of studies was based on a clearly defined search strategy. In addition to the primary Medline and Embase searches, we identified references that were cited by the articles identified in the primary database searches. Many of these secondary references directly investigated the relation between benzene exposure and cancer risk with leukemia being the main cancer. Two observers independently checked the eligibility of the studies according to *a priori *set inclusion and exclusion criteria, and identified the most appropriate effect or prevalence estimates. There was little disagreement between the two observers and these were settled by discussion. Incompatibility of the exposure or outcome criteria with our preset criteria was the main reason for exclusion.

Duplicate reports of studies were rejected and the study with the longest follow-up period or the most recent study of the cohort were chosen. All studies providing sufficient information on the relation between work exposure to benzene and leukemia were included, irrespective of whether this question was their primary or secondary objective, as measuring benzene alone was very unlikely due to fact that other chemicals were often present in the workplace alongside. The references of all included and excluded studies were further screened to identify any relevant papers. The definitions of the outcomes were based on the codes of the International Classification of Diseases (ICD) Revision 10 as follows any leukemia (C91-95), acute lymphocytic leukemia (C91.0), chronic lymphocytic leukemia (C91.1), acute myeloid leukemia (C92.0) and chronic myeloid leukemia (C92.1). A total of 15 papers which provided 16 effect estimates for the risk of leukemia in relation to benzene exposure were selected. Of these three studies applied codes of ICD revision 8, ten studies used revision 9, one revision 8 onwards, and one revision 6-9. There were no studies reporting classifications based on ICD-10 although it was available for use from 1992.

### Data extraction

Two co-authors (AK, JJ) independently examined the papers and identified and recorded the main characteristics of the study including: (1) author(s) with the year of publication; (2) study design; (3) size of study population; (4) study group; (5) geographical location; (6) time window of exposure; (7) exposure assessment; (8) study outcome; (9) effect estimate for given exposure category; (10) study selection criteria; (11) comparability in terms of confounders accounted for in the studies, for example smoking, age, socio-economic status; (12) the outcome for cohort studies and the exposure ascertained for case-control studies; and (13) the overall quality of the based on (10), (11) and (12). We defined the categories for cumulative exposure on as low from > 0 to < 40, medium from 40 to < 100 and high 100+ parts per million (ppm)-years. The two sets of data were then grouped together to identify any discrepancy in recording of the findings, and such discrepancies were then reviewed and re-assessed for the final recording.

### Assessment of study quality

We applied the Newcastle-Ottawa Scale (NOS) to assess the quality of the specific studies. The NOS for cohort and case-control studies includes the following items: 1) representativeness of the exposed cohort/adequacy of case definition; 2) selection of the non-exposed cohort/representativeness of the cases; 3) ascertainment of exposure/selection of controls; 4) demonstration that outcome of interest was not present at start of study/definition of controls; 5) comparability of cohorts on the basis of the design or analysis/comparability of cases and controls on the basis of the design or analysis; 6) assessment of outcome/ascertainment of exposure; 7) sufficiency of follow-up for outcomes to occur/similarity of method of ascertainment for cases and controls; and 8) adequacy of follow-up of cohorts/non-response rate. A star can be awarded for good quality for each item (except 1-2 stars for item 5) resulting in a range of 0-9 stars, more stars indicating higher quality.

### Statistical methods

We first calculated summary effect estimates for the four outcomes (Leuk, AML, CLL, CML) by using both the fixed-effects and random-effects models. The fixed-effects model applied the general variance-based method with inverse variances of individual study effect estimates as weights [3]. The random-effects model applied the method of DerSimonian and Laird [3]. The natural log of the effect estimates and its standard error were calculated from the effect estimates and confidence intervals (CI) presented in the articles. We ran the Stata version 10 for the fixed- and random-effects models by using the "meta" command. The Q statistics and subgroup analysis were then applied to address potential heterogeneity between study-specific effect estimates. Finally, we conducted a dose-response analysis in a meta-regression model of ln(effect estimate) by average cumulative exposure in the exposure category.

## Results

### Studies

The Medline and Embase search identified a total of 466 articles. We screened the abstracts, and excluded 287 as being clearly irrelevant or duplicates of the same study. The remaining 179 abstracts were then evaluated using *a priori *inclusion criteria (see Methods). A total of 14 articles met the selection criteria for inclusion and 165 were excluded. The reasons for exclusion were: no information on the relation of interest (n = 121) and/or no quantitative effect estimate or sufficient figures to calculate an effect estimate (n = 29) and/or duplicate publication of the same data (n = 7). Some studies provided no information on cumulative exposure to benzene (n = 8). The included articles cited additional 23 seemingly relevant articles of which one was included. The meta-analysis was based on 15 articles with 16 effect estimates summarized in Additional File 1: Table S1. Similar review produced 8 articles with 9 effect estimates for AML, 10 for CLL, 6 for CML and no articles for ALL. These fifteen studies were grouped according to the weighted average of the cumulative exposure. Additional file 2 lists the studies cited in the narrative systematic review by Schnatter et al. [2] but not included in the present meta-analysis.

### Design characteristics

From the 15 included studies, 10 were published in 1996-2004, [4-14] and the remaining five were published more recently in 2005-2008 (Additional File 1:Table S1) [15-19]. A total of 12 studies were cohort studies, and the remaining three were case-control studies. Seven studies were carried out in Europe (United Kingdom, Netherlands Sweden, Norway, Italy), one in Canada, five in the United States of America, one in China, and one in Australia. Additional File 1: Table S1 shows the workplace settings where the benzene exposure took place.

### Exposure assessment and effect estimates

The exposure assessment of 9 studies was based on workplace exposure measurements and/or job exposure matrix. Three studies used work histories and/or benzene air concentrations. The remaining three studies defined exposure on the basis of employment in a given industry, and compared cancer mortality between the industry and general population. A total of 9 studies presented cumulative exposure.

Ten studies provided effect estimates in relative risks and odds ratios and five studies presented SMRs. SMRs were converted into relative risks to provide uniform estimates of the effect size (ES) for the meta-analysis. The effect estimates from the studies varied considerably from ES of 0.96 (95% CI, 0.20-4.67) to ES of 11.3 (95% CI, 2.85-45.1). Most studies presented effect estimates for several different cancer types, however only effect estimates for "any leukemia", AML, CLL and CML were extracted for this analysis.

### Benzene exposure and the risk of any of leukemia

**Figure 1:**
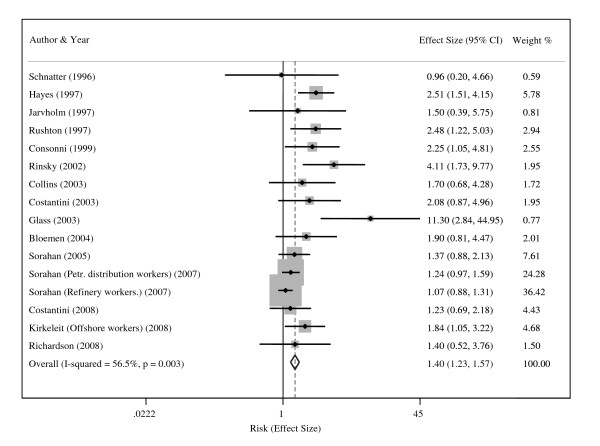
**Forest plot showing the studies providing an estimate of the relation between exposure to benzene and the risk of any leukemia**. The overall effect estimate is from the fixed-effects model.

Additional File 1: Table S1 illustrates the study-specific effect estimates for any leukemia, as well as for the three leukemia subgroups used in the meta-analysis. Nine studies provided effect estimates based on cumulative exposure to benzene, which were categorized in to low, medium, and high exposure. The remaining five studies presented SMRs comparing mortality rates between exposed cohorts and general population. Figure 1 shows a forest plot of all the study-specific effect estimates, the weights of the studies, and the summary effect estimate with the 95% confidence interval. Additional File 3: Table S3 presents the summary-effect estimates based on all 15 available studies (16 estimates), 9 studies with cumulative exposure categories, and 5 studies without quantitative exposure information.

In the fixed-effects model the summary effect size for benzene exposure was 1.40 (95% CI, 1.23-1.57), indicating a significantly increased risk of leukemia. However, both the I^2 ^index (56.5%) and Q statistics (34.47) revealed strong heterogeneity between the study-specific estimates (Additional File 3: Table S3). The random-effects model that allowed for heterogeneity yielded a summary ES of 1.72 (95% CI, 1.37-2.17). Additional File 3: Table S3 shows also summary-effect estimates for three levels of exposure, low (based on 8 studies), medium (6 studies), and high exposure (7 studies). Taking into account the average level of cumulative exposure in each study practically eliminated heterogeneity, so the variable exposure levels seemed to explain the heterogeneity observed in the overall estimate. The summary-effect estimates for low (1.64, 95% CI 1.13-2.39), medium (1.90, 95% CI 1.26-2.89), and high exposure (2.62, 95% CI 1.57-4.39) showed a clear dose-response pattern. The summary-effect estimate based on studies providing no dose information was slightly lower, 1.25 (95% CI 1.09-1.44).

To further elaborate the dose-response pattern we fitted a meta-regression model for ln(effect estimate) by average cumulative exposure to benzene. There were several effect estimates for different contrasts: eight estimates for low vs. reference, six for medium vs. reference and seven for high vs. reference category. The meta-regression model showed a moderate, statistically significant association with the R-squared value of 37% and P value of < 0.05.

**Figure 2:**
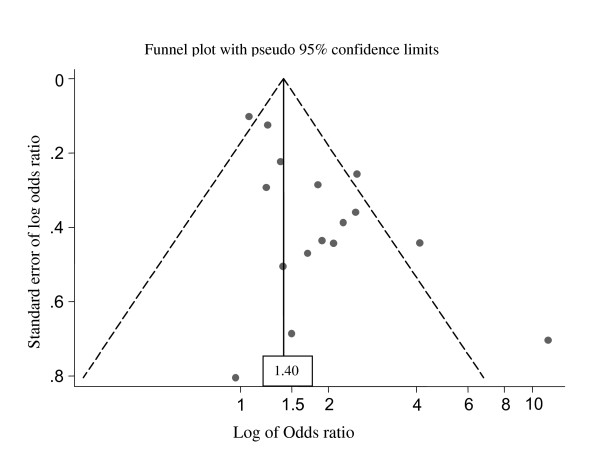
**Funnel plot showing the effect estimates (ln OR) by their standard errors (SE of ln OR)**. The vertical line indicates the summary effect estimate (1.40) from the fixed-effects model, and the dashed lines show pseudo 95% confidence limits for the summary effect estimate.

The potential for publication bias was assessed by producing a funnel plot shown in Figure 2 The vertical line indicates the summary-effect estimate from the fixed-effects model (1.40), and the corresponding pseudo 95% confidence limits converging as a function of the standard error (SE) of the effect estimate. The smaller studies with large SEs of ln OR seem to be scattered symmetrically around the summary-effect estimate, whereas the funnel plot shows substantial heterogeneity among the large studies with small SEs, with an imbalance toward large positive effect estimate. The pattern differs from a typical publication bias, in which the effect estimate from the small studies would be biased towards large positive values.

### Benzene exposure and the risk of acute myeloid leukemia (AML)

**Figure 3:**
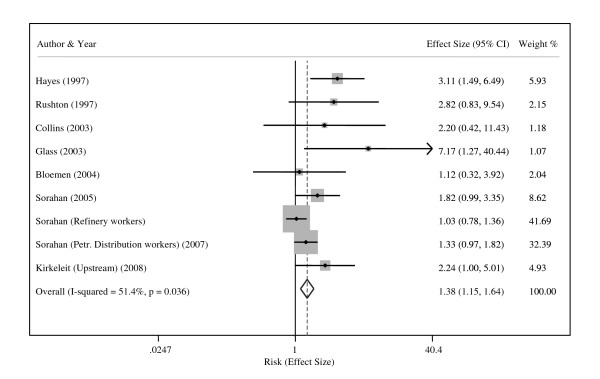
**Forest plot showing the studies providing an estimate of the relation between exposure to benzene and the risk of acute myeloid leukemia**. The summary effect estimate is from the fixed-effects model.

The study-specific effect estimates for the relation between benzene exposure and the risk of AML appear in Additional File 1:Table S1. Additional File 3: Table S3 summarizes the results of the meta-analysis on AML. In the main analysis based on 9 articles, the fixed-effects model yielded a summary-effect estimate of 1.38 (95% CI, 1.15-1.64), and the study-specific effect estimates were homogeneous (I^2 ^index 51.4%, Q statistic of 16.46, P 0.036) (Figure 3). Four studies provided information on dose, and the dose-specific effect estimates were homogeneous and presented a clear dose-response pattern (low: 1.94, 95% CI 0.95-3.95; medium 2.32, 95% CI 0.90-5.94; high: 3.20, 95% CI 1.09-9.45).

The meta-regression model for AML was based on four effect estimates for low vs. reference category, two for medium vs. reference and two for high vs. reference category. The model for the relation between cumulative exposure to benzene and the risk of AML showed no association (R-squared value of 3% and P value 0.813).

### Benzene exposure and the risk of chronic myeloid leukemia (CML)

The summary-effect estimate for CML was 1.05 (95% CI, 0.83-1.34), and the study-specific estimates were homogeneous. There were no studies applying cumulative exposure. The Egger's statistics did not indicate any publication bias (P value 0.57).

### Benzene exposure and the risk of chronic lymphocytic leukemia (CLL)

A total of 10 study-specific effect estimates yielded a summary-effect estimate of 1.31 (95% CI, 1.09-1.57). There was no indication of heterogeneity, and the random-effects model produced similar results (Additional File 3: Table S3). Six studies provided effect estimates based on cumulative exposure (dose). The summary-effect estimate for low exposure was 1.83 (95% CI 0.75-4.48), for medium exposure 1.67 (0.86-3.24), and for high exposure 3.50 (0.90-13.2), the latter was based on only one study available. There was no indication of publication bias (Egger's statistics: P value 0.06).

## Discussion

This systematic review and meta-analysis based on 15 available epidemiologic studies provides evidence of an association between benzene exposure at work and leukemia risk. The summary estimate from the fixed-effects model was 1.40 (95% CI 1.23-1.57), but the study-specific estimates were heterogeneous. Focusing on 9 studies that provided information on cumulative exposures and stratifying the effect estimates according to the magnitude of cumulative exposure eliminated the heterogeneity. The summary-effect estimate was 1.64 (1.13-2.39) for low, 1.90 (1.26-2.89) for medium, and 2.62 (1.57-4.39) for high exposure, showing evidence of a dose-response relation. The summary effect estimate for the studies which did not have dose information was lower 1.25 (1.09-1.44). Also the meta-regression model was consistent with a dose-response pattern. The results provided some evidence of an increased risk for AML and CLL. The meta-analysis indicated consistently a lack of association between benzene exposure and the risk of CML. There was not sufficient information on ALL.

The outcome assessment in all the specific studies was based on an ICD-diagnosis. Although there was a significant association between exposure to benzene and the broad category of any leukemia (ICD C91-95), there was substantial heterogeneity in the effects on specific leukemia ranging from a strong summary effect for AML to no effect for CML. Our results indicate that the use of the broad category of any leukemia underestimates the magnitude of the effect on AML. Although the summary-effect estimates for any leukemia, as well as for AML and CLL indicated an increased risk, the study-specific effect estimates presented strong heterogeneity.

We were able to retrieve some type of quantitative estimate for cumulative exposure to benzene from 9 studies. Additional File 1: Table S1 displays estimates of cumulative exposure for different exposure categories. Although exposure assessment varied between the studies, each study applied similar approaches to different levels of exposure. Use of exposure categories based on cumulative exposure reduced or practically eliminated this heterogeneity, suggesting that different amounts of benzene exposure in different studies explained the heterogeneity observed in the overall risk estimates. For example, for any leukemia the effect estimate for better quality studies (NOS 6-9) was 1.32 (95% CI 1.15-1.51), and for others (NOS 0-5) 1.79 (1.34-2.38). The summary-effect estimates for studies without dose information were presented mainly as standardized mortality ratios using external cancer mortality rates as the reference group. Their estimates were systematically lower than those from the studies providing data for dose-response analyses. A funnel plot analysis of studies on benzene exposure and leukemia risk did not show any suggestion of publication bias [20].

Several studies have been published since the most recent systematic reviews [2,21,22] on benzene and leukemia, and ours is to our knowledge the first meta-analysis on this topic.

In 1989, Lamm and colleagues published a risk assessment based on a large cohort study conducted by NIOSH (including 9 cases of leukemia), and compared their results with those of the other available large studies [21]. They concluded that AML can be caused by excessive benzene exposure, meaning a peak benzene exposure greater than 20 ppm or an estimated cumulative benzene exposure greater than 250 ppm-years. This finding was consistent across the reviewed studies except a Chinese study by Wong. This early review reported no consistent evidence for ALL, CML, or CLL in relation to benzene exposure. In 1997, Savitz and Andrews reviewed epidemiologic research on lymphatic and hematopoietic cancers. They identified 14 studies, three community-based and 11 industry-based, on benzene and total leukemia and 16 studies, nine community-based and seven industry-based, on benzene and specific histologic types of leukemia [22]. However, they did not conduct any meta-analyses. They concluded that the "epidemiologic evidence linking benzene to leukemia in the aggregate, as well as acute and chronic lymphocytic and myeloid leukemia, is no less persuasive than that for AML alone", but did not suggest any quantitative estimates.

In the most recent systematic review published in 2005, Schnatter and colleagues assessed 22 industry-based cohort and case-control studies. A high and significant AML risk was reported across study designs, especially in more highly exposed workers of rubber, shoe, and paint industry. Results on CLL were controversial with an increased risk in nested case-control studies, but with no increase in cohort studies. Data for ALL and CML were deemed sparse and inconclusive [2].

The results of our systematic review both strengthen the evidence of the effect of benzene exposure on leukemia risk, and provide quantitative estimates of effect size. We detected substantial heterogeneity between the different types of leukemia, which reduces the relevance of the overall estimate. Thus we also assessed the leukemia-specific effect sizes. The risk of AML was estimated to be two-fold for cumulative exposure below 40 ppm-years, 2.3-fold for exposures from 40 ppm-years to below 100 ppm-years, and over 3-fold for exposures 100 ppm-years and above. These estimates indicated an increased risk related to substantially lower dose than that suggested by Lamm and colleagues [21]. As a new contribution, our results also show that the available evidence is consistent with no effect on CML. Our results strengthen the evidence that benzene exposure also increases the risk of CLL, suggesting a dose-response pattern, although the effect estimate for the highest exposure category is based on a single study. Consistently with the previous reports, we found that there is no sufficient evidence to make any inference on the effects of benzene exposure to ALL.

## Conclusions

Our study provides consistent evidence that exposure to benzene at work increases the risk of leukemia with a dose-response pattern. The results showed some evidence of an increased risk for AML and CLL. The meta-analysis indicated consistently a lack of association between benzene exposure and the risk of CML. The evidence was insufficient to make any inference on the effects on ALL. For the purposes of clinical, occupational health, and policy implications, it is important to note that a significantly increased risk of any leukemia and AML was observed already in relation to the low benzene exposure and that the risk varied according to the type of leukemia.

In 1946, The American Conference of Governmental Industrial Hygienists set the first occupational exposure limit for benzene to 325 mg/m^3 ^(100 ppm), and in 1963 the limit was reduced to 35 ppm. Currently most European and North American countries have harmonised the limit to 1.63-3.25 mg/m^3 ^(0.5-1 ppm) This recent figure was agreed within the European Union in 1997 and was adopted within standard setting committee [23].

## Abbreviations

ALL: Acute lymphocytic leukemia; AML: Acute myeloid leukemia; CLL: Chronic lymphocytic leukemia; CML: Chronic myeloid leukemia; CIR: Cumulative incidence ratio; ICD: International Classification of Diseases; OR: Odds ratio; NOS: Newcastle-Ottawa Scale; RR: Relative risk; SIR: Standardized incidence rate ratio; SRR: Standardized relative rate; SMR: Standardized mortality ratio.

## Competing interests

The authors declare that they have no competing interests.

## Authors' contributions

AK conducted the literature search, reviewed the articles, conducted the statistical analyses, and drafted the manuscript. MSJ and EP made substantial contributions to interpretation of data, and were involved in drafting the manuscript or revising it critically for important intellectual content. JJKJ conceived and designed the study, reviewed the articles, and supervised the work in all phases. All authors read and approved the final manuscript.

## Supplementary Material

Additional file 1 **Table S1**. Design characteristics of studies included in the meta-analysis Click here for file

Additional file 2 **Table S2**. Studies not included and the reasons for exclusion Click here for file

Additional file 3 **Table S3**. Summary of effect size for the relation between benzene exposure and risk of leukaemia and dose-response analysis Click here for file
