# Supplementary material for: Exposure to benzene at work and the risk of leukemia: a systematic review and meta-analysis
Source: Environ Health. 2010 Jun 28;9:31. doi: 10.1186/1476-069X-9-31 (PMC2903550; doi:10.1186/1476-069X-9-31)
Supplement: Additional file 1 — Table S1. Design characteristics of studies included in the meta-analysis [file 1476-069X-9-31-S1.DOC]

##

##

##

## 
