# Supplementary material for: Exposure to benzene at work and the risk of leukemia: a systematic review and meta-analysis
Source: Environ Health. 2010 Jun 28;9:31. doi: 10.1186/1476-069X-9-31 (PMC2903550; doi:10.1186/1476-069X-9-31)
Supplement: Additional file 2 — Table S2. Studies not included and the reasons for exclusion [file 1476-069X-9-31-S2.DOC]

**Studies included by Schnatter et al. [2], but excluded from the present meta-analysis**

| **Study** | **Reason not used in study** | **Study group** | **Geographic location** |
| --- | --- | --- | --- |
| Vigliani (1964) | Individual Case reports | Patients exposed to benzene | Italy |
| Aksoy (1974) | No risk measure | Shoe workers exposed to benzene | Italy |
| Linos (1980) | Very limited benzene data | Residents in Olmsted County | USA |
| DeCoufle (1983) | Estimated risk rather than calculated | Chemical workers exposed to benzene and other agents | USA |
| Tsai (1983) | No leukemia cases | Refinery workers | Texas |
| Flodin (1986) | No Benzene exposure | Radiation, electrical workers | Sweden |
| Linet (1987) | Limited occupational history attained from census data | CLL diagnosed patients | Baltimore |
| Malone (1989) | Heavily reliant on questionnaire | Population based case control study | USA |
| Crane (1992) | Environmental exposures alongside occupational | Patients newly diagnosed with AML | Texas |
| Richardson (1992) | Haematological records for over 30 year olds not all diagnosed | Many patients hospitalized in clinical department | France |
| Ciccone (1993) | Concentrating on chromosome aberrations | Patients from Torino Hospital | Italy |
| Crump (1994) | Overlap with Rinsky 2002 | Pilofilm cohort | Ohio |
| Mele (1995) | Environmental exposures alongside occupational | Multicentre case control study | Italy |
| Li (1997) | Does not satisfy inclusion criteria of only English papers | Workers in 12 cities | China |
| Lynge (1997) | Occupational exposure to exhaust fumes were not included as many people were exposed to gasoline | Service station workers | Norway, Denmark, Sweden and Finland |
| Albin (2000) | Concentrating on chromosome aberrations | Patients from Lund Hospital | Sweden |
| Guenel (2002) | No exposure data available, therefore mostly based on assumptions | Workers exposed to benzene in gas and electric occupations | France |
| Adegoke (2003) | Heavily reliant on interview | Residents in Shanghai | China |
