# Supplementary material for: Exposure to benzene at work and the risk of leukemia: a systematic review and meta-analysis
Source: Environ Health. 2010 Jun 28;9:31. doi: 10.1186/1476-069X-9-31 (PMC2903550; doi:10.1186/1476-069X-9-31)
Supplement: Supplementary file 3 [file 1476-069X-9-31-S3.DOC]

Table S3- Summary of effect size for the relation between benzene exposure and risk of leukemia and dose-response analysis

	Model		Heterogeneity statistics			
	Fixed-effects model (ES [95% CI])	Random-effects model (ES [95% CI])	I^2 Index (%)	Q	(n)	*P* value
**Leukemia**						
Single stratum including total population	1.40 (1.23-1.57)	1.72 (1.37-2.17)	56.5	34.47	(16)	0.003
Dose response analysis						
Low (>0 to < 40 ppm-years)	1.64 (1.13-2.39) [66 cases*]	1.64 (1.13-2.39)	0.0	4.47	(8)	0.725
Medium (40 to < 100 ppm-years)	1.90 (1.26-2.89) [44 cases*]	1.90 (1.26-2.89)	0.0	4.23	(6)	0.516
High (100 - ppm-years)	2.62 (1.57-4.39) [33 cases*]	2.62 (1.57-4.39)	0.0	1.27	(7)	0.973
No dose available	1.25 (1.09-1.44)	1.50 (1.12-2.02)	63.5	16.44	(7)	0.012
**Acute myeloid leukemia**						
Single stratum including total population	1.38 (1.15-1.64)	1.70 (1.22-2.36)	51.4	16.46	(9)	0.036
Dose response analysis						
Low (>0 to < 40 ppm-years)	1.94 (0.95-3.95) [18 cases*]	1.94 (0.95-3.95)	0.0	1.27	(4)	0.737
Medium (40 to <100 ppm-years)	2.32 (0.91-5.94) [8 cases*]	2.32 (0.70-7.72)	38.7	1.63	(2)	0.202
High (100 - ppm-years)	3.20 (1.09-9.45) [12 cases*]	3.20 (1.09-9.45)	0.0	0.24	(2)	0.624
No dose available	1.28 (1.06-1.54)	1.48 (1.04-2.11)	57.3	9.37	(5)	0.053

**Chronic lymphocytic leukemia**						
Single stratum including total population	1.31 (1.09-1.57)	1.31 (1.09-1.57)	0.0	8.86	(10)	0.450
Dose response analysis						
Low (0< 40 ppm-years)	1.83 (0.75-4.48) 13 cases*	1.86 (0.71-4.86)	13.1	1.15	(2)	0.283
Medium (40- 99 ppm-years)	1.67 (0.86-3.24) 15 cases*	1.67 (0.86-3.24)	0.0	0.23	(3)	0.893
High (100 - ppm-years)	3.50 (0.90-13.2) 9 cases*	N/A			(1)	
No dose available	1.24 (1.02-1.50)	1.24 (1.02-1.50)	0.0	4.79	(6)	0.442
**Chronic myeloid leukemia**						
Single stratum including total population	1.05 (0.83-1.34)	1.05 (0.83-1.34)	0.0	1.69	(6)	0.890
No dose available	1.06 (0.83-1.35)	1.06 (0.83-1.35)	0.0	1.54	(5)	0.819

*Number of cases
